# Early rhythm control vs. rate control in atrial fibrillation: A systematic review and meta-analysis

**DOI:** 10.3389/fcvm.2023.978637

**Published:** 2023-02-06

**Authors:** Shaojie Han, Ruikun Jia, Zhifu Cen, Ran Guo, Shenyu Zhao, Yixuan Bai, Min Xie, Kaijun Cui

**Affiliations:** ^1^Department of Cardiology, West China Hospital, Sichuan University, Chengdu, China; ^2^Department of Cardiology, Chengdu Seventh People’s Hospital, Chengdu, Sichuan, China

**Keywords:** atrial fibrillation, early rhythm control, rate control, meta-analysis, cardiovascular outcome

## Abstract

**Objective:**

It has long been debated whether rhythm control vs. rate control strategies have differing effects on mortality and morbidity for atrial fibrillation (AF). Recently, several randomized controlled studies (RCTs) and observational trials described that an early rhythm management method was linked to a lower likelihood of negative clinical outcomes in individuals with AF. We wanted to see if an early rhythm management method may help patients with AF.

**Methods:**

We performed a systematic search to retrieve studies assessing the outcomes of early rhythm control vs. rate control in AF by using PubMed, Web of Science, Cochrane Library, and Embase published between 01/01/2000 and 15/04/2022.

**Results:**

Finally, two RCTs, one retrospective analysis of RCTs, and four observational studies were identified. Compared with rate control, early rhythm control has been linked to lower all-cause mortality. [risk ratio (RR), 0.76; 95% CI 0.69–0.83; *P* < 0.00001; *I*^2^ = 77%]. The early rhythm control group was also associated with a lower risk of cardiovascular mortality (RR, 0.68; 95% CI 0.63–0.74; *P* < 0.00001; *I*^2^ = 33), stroke (RR, 0.77; 95% CI 0.67–0.87; *P* < 0.001; *I*^2^ = 64), and heart failure hospitalization (RR, 0.74; 95% CI 0.59–0.93; *P* = 0.0009; *I*^2^ = 93%). We found no significant difference in nights spent in hospital per year, acute coronary syndrome, major bleeding, and cardiac arrest/ventricular arrhythmia between the groups.

**Conclusion:**

In this meta-analysis, early rhythm therapy was linked to a lower risk of all-cause mortality, cardiovascular mortality, stroke, and heart failure hospitalization compared with the rate control group.

**Systematic review registration:**

https://www.crd.york.ac.uk/PROSPERO/, identifier CRD42022333592.

## 1. Introduction

Atrial fibrillation (AF) is a kind of cardiovascular disease that affects millions of people throughout the world and is associated with an increased risk of mortality and morbidity, with a fivefold increased risk of stroke (1–3). The current two essential aims of AF clinical care are (1) thromboembolism prophylaxis with anticoagulation and (2) maintenance of an appropriate heart rate or sinus rhythm by medications or interventional procedures (4). Rate control is part of AF management and can adequately improve related symptoms. Rhythm control refers to the use of antiarrhythmic drugs, cardioversion, and AF ablation to try to restore and maintain sinus rhythm. It has been argued for a long time whether rhythm vs. rate control strategies have differing effects on mortality and morbidity for AF. The choice of rhythm or rate control in current guidelines relies on several randomized controlled studies (RCTs) (5–7). No significant difference in all-cause mortality, cardiovascular mortality, and other related morbidities was found between rhythm control and rate control in the meta-analyses of the above studies included (8, 9). However, the treatment of AF has changed dramatically since the above RCTs were published. Several studies have recently shown that the incidence of adverse cardiovascular outcomes was reduced by early rhythm control compared with rate control (10–17). To determine whether early rhythm control is better than rate control in patients with AF, we performed a systematic review and meta-analysis.

## 2. Materials and methods

The meta-analysis was performed following the Preferred Reporting Items for Systematic Reviews and Meta-Analyses (PRISMA) guidelines.

### 2.1. Literature search

From 1 January 2000, to 15 April 2022, a systematic search for RCTs and observational studies was undertaken using PubMed, EMBASE, Web of Science, and Cochrane Library databases, with no language restrictions. A manual search was conducted that included all of the relevant references following the computerized search. The following were among the most important search topics and terms: (1) atrial fibrillation, (2) rate control, and (3) rhythm control. Detailed search strategies are summarized in the Supplementary material. The PRISMA statement was followed when performing this meta-analysis. The review protocol has been registered in PROSPERO (registration number: CRD42022333592).

### 2.2. Selection and data abstraction

The following criteria were used to choose articles: (1) observational studies or RCTs that included patients with AF based on early rhythm control vs. rate control; (2) based on “Early Treatment of Atrial Fibrillation for Stroke Prevention Trial” (EAST-AFNET 4) study, patients were enrolled within 1 year after the first diagnosis of AF (early AF); (10) (3) the studies’ follow-up time was at least 1 year; and (4) the goal of the study was to examine the effect and prognosis of AF treated with early rhythm vs. rate control. All studies were restricted to those including human subjects who were at least 18 years old. Reviews, case studies, conference papers, comments, and animal trials were all omitted from the study. Two reviewers separately evaluated article titles and abstracts to exclude papers that were not relevant. Disagreements were addressed by consensus and, if needed, the consulting of a third reviewer. The risk of bias was assessed using the Cochrane collaboration tool for RCTs and using the Newcastle-Ottawa scale for non-randomized clinical studies. The following information was gathered from eligible studies: (1) design of the research; (2) primary/secondary outcome; (3) mean follow-up time and baseline characteristics; and (4) anticoagulation therapy, rate, and rhythm protocols. The outcomes of the present analysis were as follows: all-cause mortality, cardiovascular mortality, ischemic stroke, heart failure (HF) hospitalization, nights spent in hospital per year, acute coronary syndrome, major bleeding, and cardiac arrest/ventricular arrhythmia.

### 2.3. Statistical analysis

Dichotomous variables were investigated using the Mantel-Haenszel method. The risk ratio (RR) and 95% confidence interval (CI) were determined. Continuous variables were described as the mean and standard deviation. To examine heterogeneity, the Cochran Q and *I*^2^ statistics were utilized. We defined moderate or high heterogeneity as an *I*^2^ of more than 50%. Given the expected between-study heterogeneity, a meta-analysis was conducted using a random effects model for all outcomes. If more than 10 studies were included, a funnel plot was used to measure publication bias. By removing one study at a time, we performed a series of sensitivity analyses to establish the contribution of each study to the pooled estimate. All *P* values were two-tailed. R programming language (version 4.1.2, R Foundation) was used to perform sensitivity analyses. Review Manager Version 5.3 software (The Nordic Cochrane Centre) was used to conduct an overall effect analysis and subgroup analysis.

## 3. Results

**FIGURE 1:**
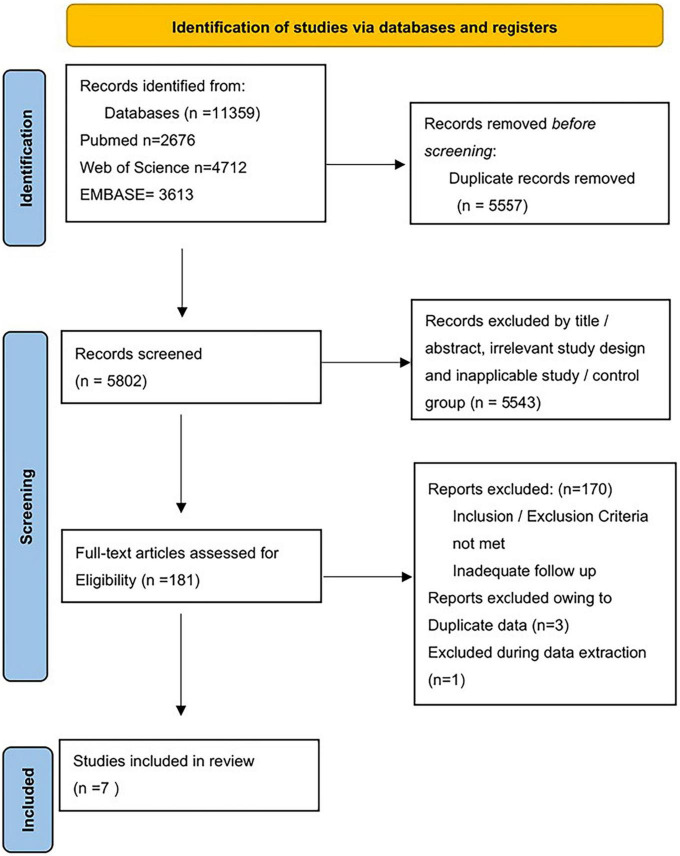
Summary of electronic search and included/excluded studies.

As shown in Figure 1, a total of 11,359 studies were found in the database. 5,557 duplicate records were excluded. A total of 5,543 records were excluded based on title/abstract, animal studies, irrelevant study design, and inapplicable study. We read the full text of 181 studies carefully. Finally, seven studies were included: two RCTs, one retrospective analysis of RCT, and four observational studies. The sample size ranged from 273 to 301,064. The mean follow-up times ranged from 1 to 5 years. Table 1 and Supplementary Table provided the features of our included studies. Table 2 summarizes the quality appraisal for the studies that were included.

**TABLE 1:** Main study characteristics.

Trials	EAST-AFNET4 (2020)	AFFIRM substudy (2021)	Kim et al. (11)	Pope et al. (17)	RAFAS trial (2022)	Proietti et al. (12)	Chao et al. (14)
Study design	RCT	RCT substudy	Retrospective	Retrospective	RCT	Retrospective	Retrospective
**Early rhythm vs. rate**							
No. of patients	1,395 vs. 1,394	1,269 vs. 1,657	9,246 vs. 7,077	6,595 vs. 37,606	178 vs. 95	2,056 vs. 1,722	62,649 vs. 238,415
**Type of AF (%)**							
First episode	38.0 vs. 37.3	NR	NR	54.8 vs. 50.6	No	22.8 vs. 32.8	NR
Paroxysmal	36 vs. 35.4	NR	NR	25.5 vs. 32.5	52.8 vs. 50.5	38.8 vs. 43.1	NR
Persistent	26 vs. 27.3	NR	NR	19.7 vs. 16.7	47.2 vs. 49.5	38.5 vs. 24.2	NR
Mean age (SD) or median (IQR)	70.2 ± 8.4 vs. 70.4 ± 8.2	69 (61–75) vs. 72 (64–78)	69 (61–75) vs. 72 (64–78)	69 (61–75) vs. 72 (64–78)	67.0 (58.0–74.0) vs. 71.0 (63.0–78.0)	68.9 ± 8.9 vs. 70.1 ± 7. 8	69 (62–76) vs. 74 (66–79)
Men (%)	53.8 vs. 53.5	60.3 vs. 59.3	52.9 vs. 51.9	58.5 vs. 55	60.7 vs. 64.2	55.9 vs. 51.0	55.52 vs. 56.56
Hypertension (%)	88.3 vs. 87.5	72 vs. 70.5	84.3 vs. 64.1	75.2 vs. 76.5	65 vs. 74.5	70.1 vs. 65.4	64.01 vs. 67.08
Valvular disease (%)	43.8 vs. 46.1	NR	8.6 vs. 10.2	NR	NR	47.2 vs. 50.3	NR
HF (%)	28.4 vs. 28.8	NR	49 vs. 54.9	23.5 vs. 21.6	6.2 vs. 9.6	NR	24.79 vs. 22.79
CAD (%)	16.9 vs. 17.2	NR	NR	25.7 vs. 24.6	6.2 vs. 6.4	21.2 vs. 22.8	7.58 vs. 8.36
NOAC	91.2 vs. 81.7 (NOAC + VKA)	NR	26.7 vs. 22.5	36.9 vs. 26.5	89.3 vs. 89.5	42.8 vs. 43	3.56 vs. 4.67
VKA		84.4 vs. 94.1	79.1 vs. 83.1	33.9 vs. 38.7	4.5 vs. 4.3	43.6 vs. 39.9	11.7 vs. 13.35
years of follow-up	median 5.1 y	median 5.1 y	median 2.1 y	mean 2 y	mean1 y	mean 675.4 d	Estimated no less than 5 years

AF, atrial fibrillation; RCT, randomized controlled trials; NR, not report; HF, heart failure; CAD, Coronary artery disease; NOAC, non-vitamin K antagonist oral anticoagulant. VKA, vitamin K antagonist oral anticoagulant; TIA, transient ischemic attack; IQR, interquartile range.

**TABLE 2:** **(A)** Quality assessment of cohort study by Newcastle-Ottawa scale.

References	Selection				Comparability	Outcome			Score
	**1**	**2**	**3**	**4**	**1**	**1**	**2**	**3**	
Kim et al. (11)	1	1	1	1	2	1	1	1	9
Pope et al. (17)	1	1	1	1	2	1	1	1	9
Proietti et al. (12)	1	1	1	1	1	1	1	1	8
Chao et al. (14)	1	1	1	1	2	1	1	1	9

Selection: 1. Representativeness of the exposed cohort; 2. Selection of the non-exposed cohort; 3. Ascertainment of exposure; 4. Demonstration that the outcome of interest was not present at start of the study. Comparability: 1 Comparability of cohorts based on the design or analysis. Outcome: 1 Assessment of outcome; 2. Was follow-up long enough for outcomes to occur? 3. Adequacy of follow-up of cohorts.

**TABLE 2:** **(B)** Quality assessment of randomized control trials by Cochrane collaboration’s tool.

Study	Random sequence generation	Allocation concealment	Blinding of participants and personnel	Blinding of outcome assessment	Incomplete outcome data	Selective reporting	Other bias
EAST-AFNET 4 (2020)	Low risk of bias	Unclear risk of bias	High risk of bias	Low risk of bias	Low risk of bias	Low risk of bias	Low risk of bias
AFFIRM substudy (2021)	Unclear risk of bias	High risk of bias	High risk of bias	Low risk of bias	Low risk of bias	Low risk of bias	High risk of bias
RAFAS trial (2022)	Low risk of bias	Unclear risk of bias	High risk of bias	Unclear risk of bias	Low risk of bias	High risk of bias	Low risk of bias

### 3.1. Mortality

**FIGURE 2:**
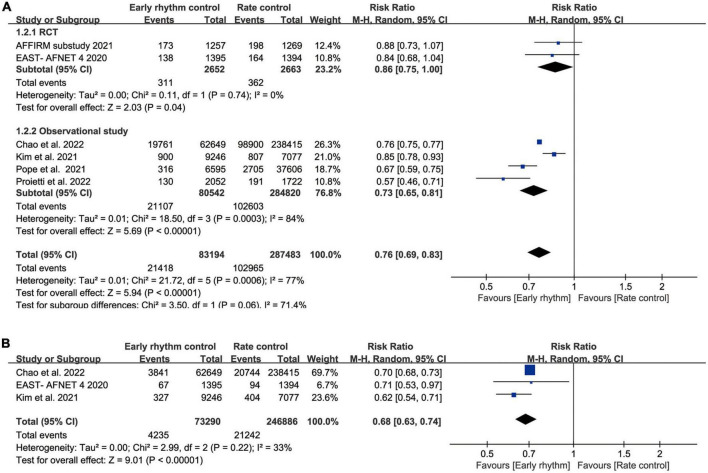
**(A)** Forest plot showing all-cause mortality between early rhythm group and rate group. **(B)** Forest plot showing cardiovascular mortality between early rhythm group and rate group.

Seven studies reported all-cause mortality (10–15, 17, 18). Mortality was as high as 50% in Ionescu-Ittu et al.’s study during a mean follow-up of 3 years. When we included this study, the heterogeneity of our analysis was 97%. Therefore, we excluded this study from the analysis to better interpret our results and reduce heterogeneity. Finally, the pooled analysis showed lower all-cause mortality in the early rhythm group than in the rate control group (RR 0.76; 95% CI 0.69–0.83; *P* < 0.00001; *I*^2^ = 77%; Figure 2A). Analysis of pre-defined subgroups based on the study design also showed significantly lower mortality with RCT (RR, 0.86; 95% CI 0.75–1.00; *P* = 0.04) or observational studies (RR, 0.73; 95% CI, 0.65–0.81; *P* < 0.00001; Figure 2A). When the data were pooled based on the time it took for patients to enroll (before 2009 vs. after 2009), similar results were found (Supplementary Figure 1). Three clinical studies reported cardiovascular mortality (10, 11, 14). The incidence of cardiovascular mortality was low in early rhythm control compared to rate control (RR, 0.68; 95% CI 0.63–0.74; *P* < 0.00001; Figure 2B) without statistically significant heterogeneity (*I*^2^ = 33%).

### 3.2. Morbidity

**FIGURE 3:**
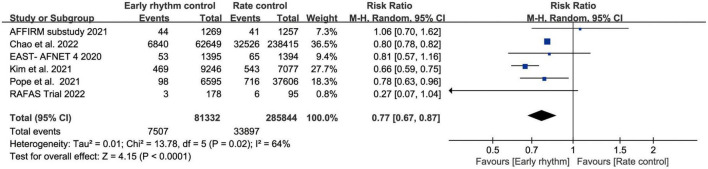
Forest plot showing risk of stroke between early rhythm group and rate group.

**FIGURE 4:**
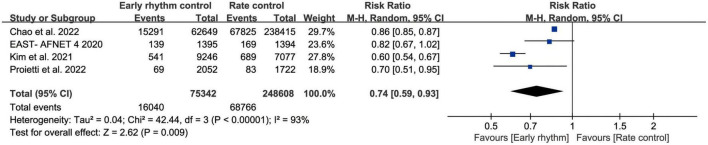
Forest plot showing risk of heart failure hospitalization between early rhythm group and rate group.

Six studies assessed ischemic stroke (10, 11, 13–15, 17). Early rhythm control was linked to a lower risk of stroke in the patients (RR, 0.77; 95% CI 0.67–0.87; *P* < 0.0010; *I*^2^ = 64%; Figure 3). We also performed subgroup analyses based on the time of patient enrollment, and study design and found no change in the above findings (Supplementary Figure 2). Only four studies included HF hospitalization (10–12, 14). Patients with early rhythm were associated with a reduced relative risk of HF hospitalization. However, there was obvious heterogeneity (RR, 0.74; 95% CI, 0.59–0.93; *P* = 0.0009; *I*^2^ = 93%; Figure 4).

Regarding nights spent in the hospital per year, acute coronary syndrome, major bleeding, and cardiac arrest/ventricular arrhythmia, early rhythm control and rate control showed no significant differences. The results were summarized in Table 3 and Supplementary Figure 3.

**TABLE 3:** Outcome of patients who underwent early rhythm control or rate control for atrial fibrillation.

Outcome endpoints	No. of studies	Participants	*P*-value	Effect estimate (95% CI)	*I* ^2^
All-cause mortality	7	370950	<0.01	0.76 (0.69, 0.83)	77%
Cardiovascular mortality	3	320176	<0.01	0.68 (0.63, 0.74)	33%
Stroke	6	367176	<0.01	0.77 (0.67, 0.87)	64%
Heart failure hospitalization	4	323950	<0.01	0.74 (0.59, 0.93)	93%
Nights spent in hospital per year	4	25412	0.63	−0.03 (−0.17, 0.10)	95%
Acute coronary syndrome	4	322702	0.44	0.96 (0.87, 1.06)	11%
cardiac arrest/ventricular arrhythmia	3	21638	0.21	1.18 (0.91, 1.52)	0%
Major bleeding	3	65219	0.60	0.95 (0.78, 1.16)	36%

Two studies reported adverse events related to treatment in early rhythm control vs. rate control. We did not include pooled analysis because of the limited number of studies. We found that syncope, cardiac tamponade, atrioventricular block, and pacemaker implantation were higher in the early rhythm group than rate control group, but none were statistically significant.

### 3.3. Sensitivity analyses

We conducted a sensitivity analysis by eliminating one study at a time to determine how each one affected the outcomes. The sensitivity analysis findings are summarized in Supplementary Figure 4. Overall, successive exclusion of each study had no meaningful effect on any of the clinical outcomes. Due to the limited number of included studies, we did not perform a publication bias assessment.

## 4. Discussion

This meta-analysis was performed to compare the benefits of early rhythm control vs. rate control in patients with AF. When compared to rate control, early rhythm control appears to be related to lower all-cause mortality, cardiovascular mortality, stroke, and HF hospitalization. Nevertheless, there was no difference between the two groups for acute coronary syndrome, major bleeding, nights spent in the hospital per year, and cardiac arrest/ventricular arrhythmia. To the best of our knowledge, our systematic review meta-analysis is the first to report on early rhythm control in patients with AF.

### 4.1. Interpretation of results

Our study concluded that early rhythm control may benefit patients compared with rate control. AF is usually thought to be a progressive disorder, in which arrhythmia begins as paroxysmal form and progresses from persistent to “permanent” AF with electrical and structural remodeling of the atrium (19). AF produces mechanisms for self-perpetuation after it has been established (“AF begets AF’) (20). Structural, electrical, and autonomic remodeling are all affected by arrhythmia and it can exacerbate pre-existing issues, making the patient more susceptible to recurring and chronic AF (21, 22). In addition, the Framingham Heart Study demonstrated that in the first year after AF is identified, the risk of cardiovascular problems increased (2). Amiodarone, the most effective medicine now available for long-term sinus rhythm maintenance, has anti-remodeling effects (23). In patients with AF, catheter ablation is superior to medical therapy for the maintenance and restoration of sinus rhythm (4, 24). Previous research has suggested that catheter ablation can prevent left atrial remodeling (25, 26). A shorter period between the first AF diagnosis and the ablation therapy has also been demonstrated to improve the chances of ablation success (27). Therefore, maintaining sinus rhythm as early in the natural history as feasible would appear to be a rational method to avoid AF development. However, since 2002, rhythm control has been proven to be unlikely to reduce all-cause and cardiovascular mortality in the general population compared to rate control in RCTs (5, 6, 28). This seemingly conflicting outcome might be explained. The poor rate of sinus rhythm restoration and maintenance in most of these experiments is a key issue. Only 39% of patients in the rhythm-control arm of the RACE experiment were in sinus rhythm at the end of the study (28). Patients in the late phases of the illness process were also included in these studies. Patients with chronic AF were enrolled in the STAF, PIAF, and RACE studies (28–30). Likewise, a substudy of the AFFIRM study demonstrated no difference in all-cause mortality, and ischemic stroke when comparing early rhythm control with rate control in patients with AF. Of all the studies we included, this was the only study that early rhythm control showed no benefit compared with rate control. The proportion of anticoagulants used in the early rhythm group was lower than that in the rate group (84.4 vs. 94.1%). We think that this was a key factor leading to the above results. The AFFIRM study recruited patients with dilated left atrium (65%). Even with the use of antiarrhythmic medicines, it is difficult to reverse structural abnormalities and sustain sinus rhythm once AF has structural changes. In addition, over the last 20 years, AF ablation is an important role in the treatment of AF. The AFFIRM study included patients who were not treated with catheter ablation.

### 4.2. Clinical implications

These findings imply that early rhythm control is superior to rate control. Therefore, patients with AF should receive rhythm control immediately. However, guidelines currently recommend rhythm control therapy to improve symptoms and quality of life in symptomatic patients with AF (IA) (4). In fact, many newly diagnosed AF patients may be asymptomatic (31). A new AF diagnosis is linked to a high risk of stroke (7%), heart failure (14%), and death (49%) (32). Early rhythm management has been proven to have a lower risk of death and stroke in some studies. Nevertheless, there is no recommendation in the current guidelines early rhythm management to reduce severe adverse cardiovascular events such as stroke and mortality. Although early rhythm therapy has some associated side effects, long-term antiarrhythmic drugs-related significant adverse events, and mortality are usually linked to chronic AF and structural heart disease (33). Catheter ablation, an effective strategy for rhythm control, showed no significant increase in adverse events compared with the standard care group (34). Therefore, future guidelines may support the early rhythm control management of AF based on the long-term effects in reduced all-cause mortality, cardiovascular mortality, stroke, and HF hospitalization. Furthermore, patient selection and interaction between patient and operator should not be overlooked.

## 5. Limitations

First, due to the nature of observational studies, biases cannot be eliminated. Differences in techniques, demographics, and backgrounds inevitably convey unidentified confounders. Despite the random effects method employed in quantitative analysis, heterogeneity of clinical features and interventions among trials is a significant limitation. Second, several studies included patients between 1996 and 2020, causing changes in therapy over time. The difference between the samples included in our study was large, ranging from 273 to 301,064. Therefore, we conducted a sensitivity analysis by eliminating one study at a time to determine how each one affected the outcomes. We did not find a meaningful effect on any of the clinical outcomes. Third, the lack of patient-level data made an extensive evaluation of baseline features regarding clinical outcomes impossible. However, all of the studies’ baseline parameters were well-matched in both groups. Fourth, the studies we included had different definitions of early intervention, but we performed a series of sensitivity analyses and found no significant difference. Finally, most of the studies we included were real-world studies, and only one large-scale prospective RCT in our meta-analysis. In our subgroup analysis, a retrospective analysis of an RCT was also classified as a randomized controlled study.

## 6. Conclusion

This is the first meta-analysis to conclude that early rhythm control may be more beneficial than rate control in patients with AF. Our study demonstrated that early rhythm control can reduce all-cause mortality, cardiovascular mortality, stroke, and HF hospitalization. However, early rhythm control was not associated with acute coronary syndrome, major bleeding, nights spent in the hospital per year, and cardiac arrest/ventricular arrhythmia. We hope that more research will be done in the future to confirm our findings.

## Data availability statement

The original contributions presented in this study are included in the article/Supplementary material, further inquiries can be directed to the corresponding author.

## Author contributions

SH and RJ conceived the review. SH drafted and wrote the manuscript. RJ, ZC, SZ, RG, and YB revised and edited all the version of the manuscript. During the revision of the manuscript, MX made great contributions to us, including the correction of the manuscript, statistical analysis, language polishing and literature retrieval. KC revised the sections. All authors contributed to manuscript revision and approved the submitted version.
