# Supplementary material for: Early rhythm control vs. rate control in atrial fibrillation: A systematic review and meta-analysis
Source: Front Cardiovasc Med. 2023 Feb 6;10:978637. doi: 10.3389/fcvm.2023.978637 (PMC9939510; doi:10.3389/fcvm.2023.978637)
Supplement: Supplementary file 1 [file Data_Sheet_1.docx]

# Supplementary material

**Search strategy**

#1 "atrial fibrillation"[MeSH Terms] OR "atrial fibrillation"[All Fields]

#2 “rate control” [All Fields]

#3 “rhythm control” [All Fields]

#4 #2 OR #3

#5 #1 AND #4

**Supplementary Figure**

Supplementary Figure 1


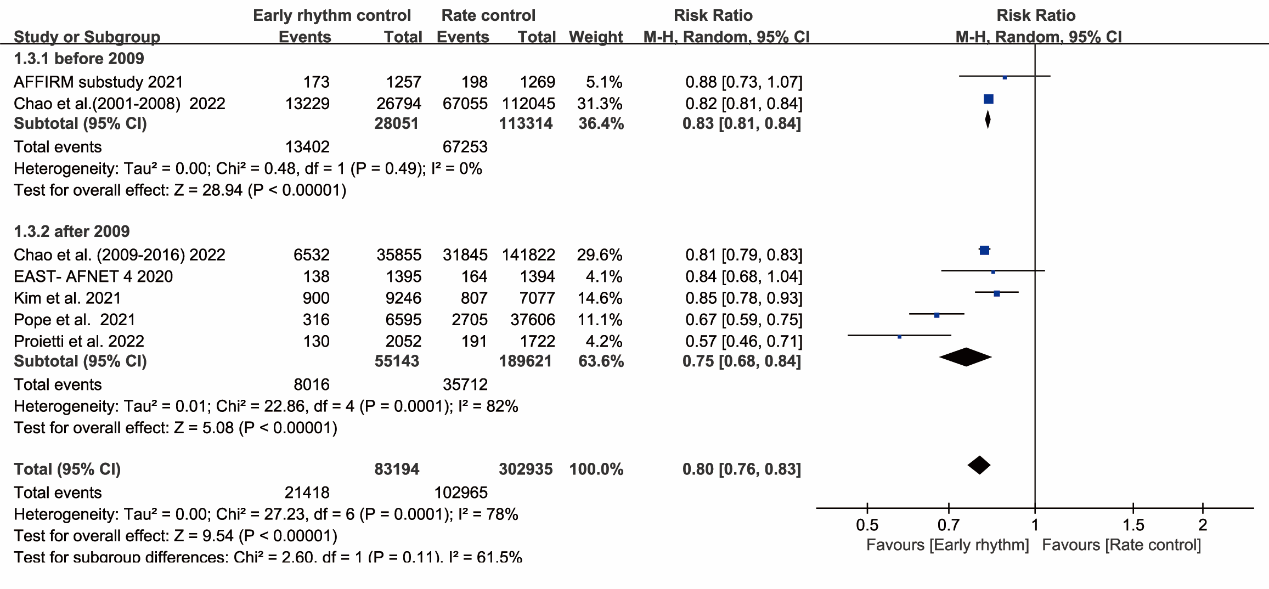


Supplementary Figure1 Subgroup analysis comparing risk of all-cause mortality between early rhythm and rate control; patient enrollment time (before 2009, after 2009).

Supplementary Figure 2

**
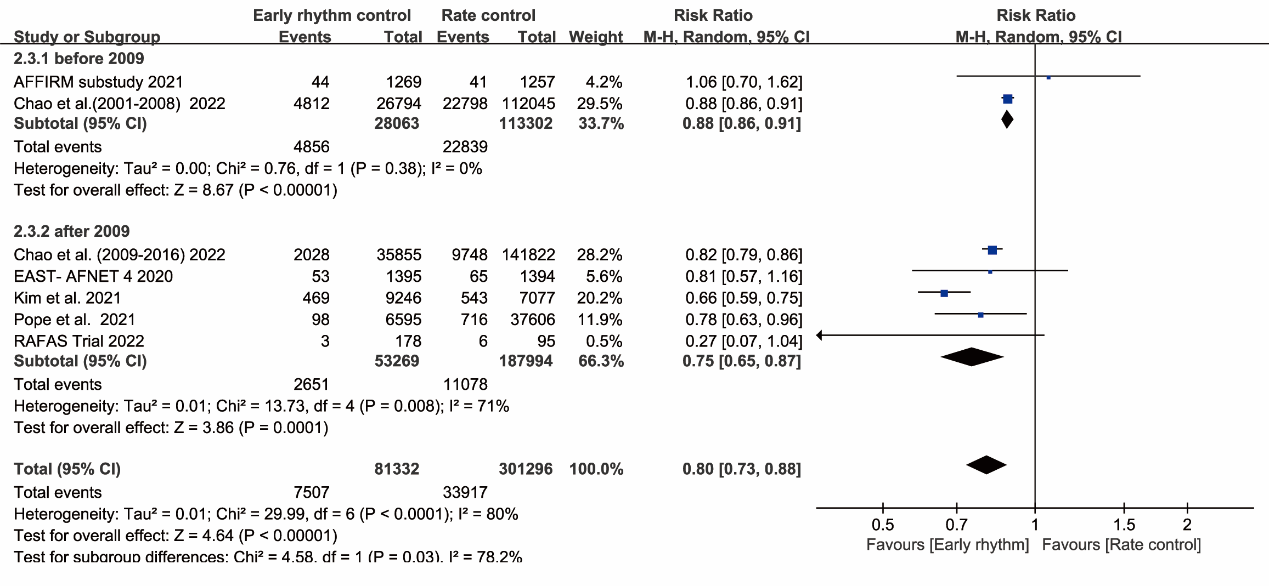
**

Supplementary Figure2 Subgroup analysis comparing risk of stroke between early rhythm and rate control; b patient enrollment time (before 2009, after 2009).

Supplementary Figure 3

**
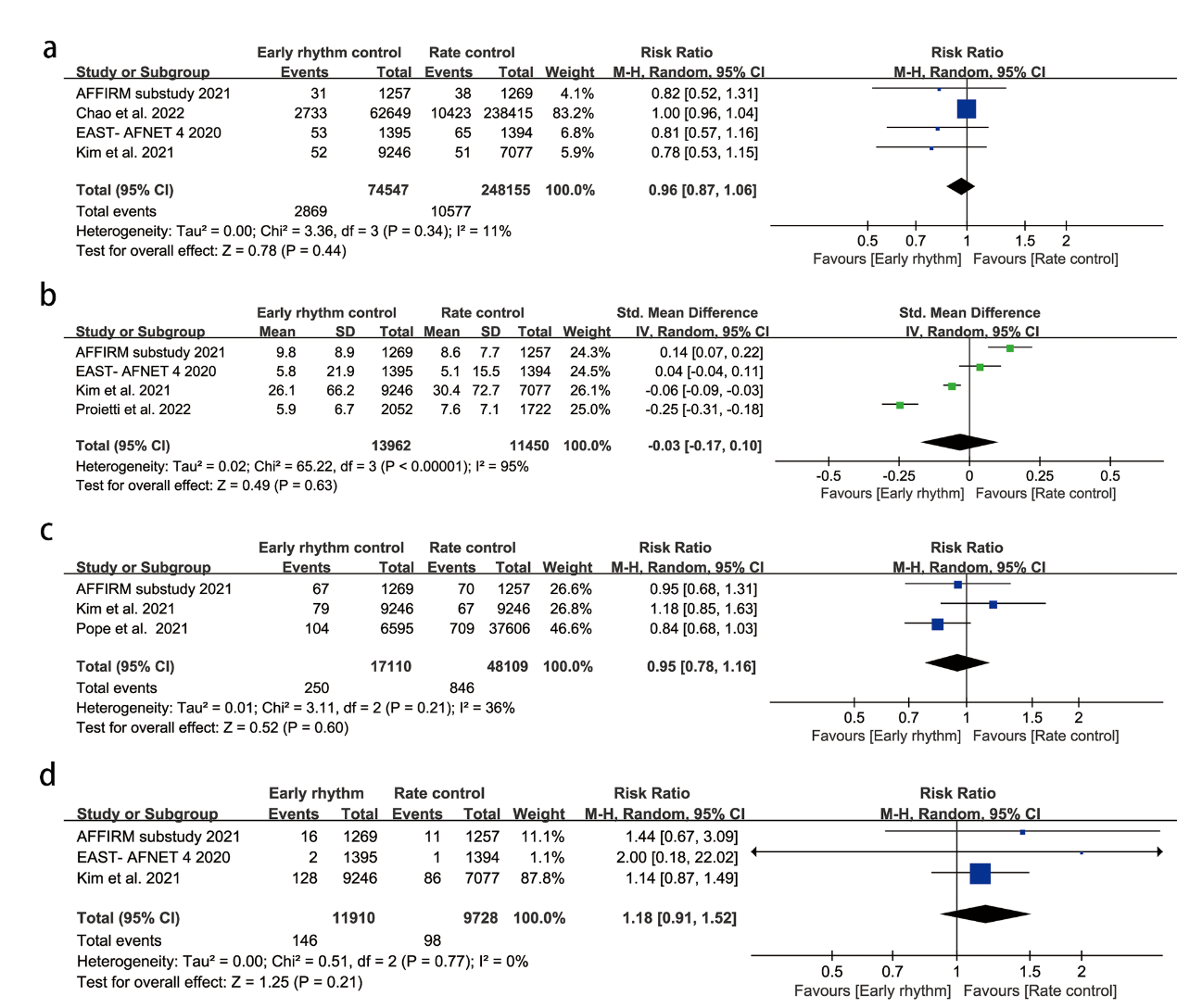
**

Supplementary Figure3 Forest plot comparing risk of acute myocardial infarction(3a), nights spent in hospital per year(3b), major bleeding(3c), cardiac arrest/ventricular arrhythmia(3d) and between rhythm and rate controls.

Supplementary Figure 4a

**

**

Supplementary Figure 4b

**

**

Supplementary Figure 4c

**
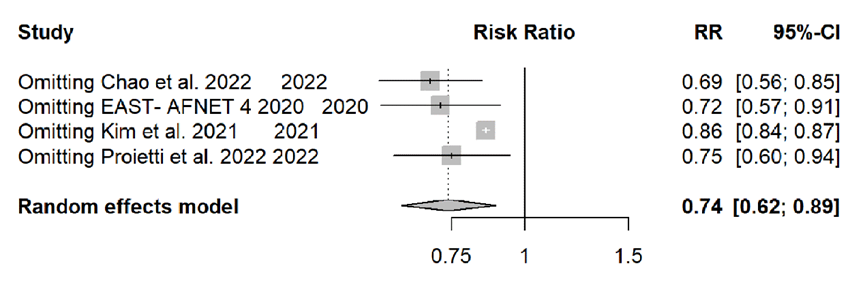
**

Supplementary Figure4 Sensitivity analysis in a random-effects model for the all-cause death(4a), stroke(4b), and heart failure hospitalization(4c) in the total publication

Supplementary Table 1 Methods in the trials comparing early rhythm control vs. rate control for atrial fibrillation

| **study** | **Definition of Early treatment atrial fibrillation** | **Rhythm intervention** | **Rate intervention** | **Primary outcome** |
| --- | --- | --- | --- | --- |
| EAST-AFNET 4(2020) | Treatment within 1 year of AF diagnosis. | antiarrhythmic drugs or atrial fibrillation ablation, as well as cardioversion | therapy that maintained the heart rate within guideline recommended targets without rhythm intervention | Death from cardiovascular causes, Stroke, Hospitalization with heart failure and acute coronary syndrome |
| AFFIRM substudy(2021) | Treatment within 6 months of AF diagnosis | Amiodarone, disopyramide, flecainide, moricizine, procainamide, propafenone, quinidine, sotalol, and combinations of these drugs. | beta-blockers, calcium-channel blockers (verapamil and diltiazem), digoxin, and combinations of these drugs. | all-cause mortality |
| Kim et al. (2021) | rhythm or rate control treatments within 1 year after its diagnosis | a prescription of more than a 90-day supply of any rhythm-control drugs in the 180-day period since the first prescription or the performance of an ablation procedure for AF. | a prescription of more than a 90-day supply of any rate-control drugs in the 180-day period since the first prescription and with no prescription of rhythm-control drug and no ablation within this period. | death from cardiovascular causes, ischaemic stroke, hospitalisation for heart failure, or acute myocardial infarction |
| Pope et al. (2021) | Treatment within 6 weeks of AF diagnosis | pharmacological or direct current cardioversion | not report | non-haemorrhagic stroke or systemic embolism, major bleeding, and all cause mortality |

Table 1. (Continued)

| study | Definition of Early treatment atrial fibrillation | Rhythm intervention | Rate intervention | Primary outcome |
| --- | --- | --- | --- | --- |
| RAFAS Trial (2022) | Treatment within 2 months of AF diagnosis | a stepwise sequential approach: AADs, electrical cardioversion, and catheter ablation | heart rate control and anticoagulation medications were prescribed; AADs, cardioversion, and catheter ablation were not performed within 2 months of an acute stroke. | Recurrent ischemic stroke within 12 months |
| Proietti et al. (2022). | rhythm or rate control treatments within 1 year after its diagnosis | electrical cardioversion, pharmacological cardioversion, catheter ablation, or were prescribed an antiarrhythmic drug (Class Ia, Class Ic, Class III) | beta blockers, digoxin, or non-dihydropyridine calcium-channel blockers | Evaluation of quality of life, Evaluation of health‑care resources use , and Major adverse events |
| Chao et al. (2022) | rhythm or rate control treatments within 1 year after its diagnosis | received antiarrhythmic drugs (AADs) or catheter ablation | similar to EAST-AFNET 4 study | ischemic stroke, heart failure hospitalizations, AMI, all-cause mortality |
